# Proposing a Principle-Based Approach for Teaching AI Ethics in Medical Education

**DOI:** 10.2196/55368

**Published:** 2024-02-09

**Authors:** Lukas Weidener, Michael Fischer

**Affiliations:** 1 UMIT TIROL – Private University for Health Sciences and Health Technology Hall in Tirol Austria

**Keywords:** artificial intelligence, AI, ethics, artificial intelligence ethics, AI ethics, medical education, medicine, medical artificial intelligence ethics, medical AI ethics, medical ethics, public health ethics

## Abstract

The use of artificial intelligence (AI) in medicine, potentially leading to substantial advancements such as improved diagnostics, has been of increasing scientific and societal interest in recent years. However, the use of AI raises new ethical challenges, such as an increased risk of bias and potential discrimination against patients, as well as misdiagnoses potentially leading to over- or underdiagnosis with substantial consequences for patients. Recognizing these challenges, current research underscores the importance of integrating AI ethics into medical education. This viewpoint paper aims to introduce a comprehensive set of ethical principles for teaching AI ethics in medical education. This dynamic and principle-based approach is designed to be adaptive and comprehensive, addressing not only the current but also emerging ethical challenges associated with the use of AI in medicine. This study conducts a theoretical analysis of the current academic discourse on AI ethics in medical education, identifying potential gaps and limitations. The inherent interconnectivity and interdisciplinary nature of these anticipated challenges are illustrated through a focused discussion on “informed consent” in the context of AI in medicine and medical education. This paper proposes a principle-based approach to AI ethics education, building on the 4 principles of medical ethics—autonomy, beneficence, nonmaleficence, and justice—and extending them by integrating 3 public health ethics principles—efficiency, common good orientation, and proportionality. The principle-based approach to teaching AI ethics in medical education proposed in this study offers a foundational framework for addressing the anticipated ethical challenges of using AI in medicine, recommended in the current academic discourse. By incorporating the 3 principles of public health ethics, this principle-based approach ensures that medical ethics education remains relevant and responsive to the dynamic landscape of AI integration in medicine. As the advancement of AI technologies in medicine is expected to increase, medical ethics education must adapt and evolve accordingly. The proposed principle-based approach for teaching AI ethics in medical education provides an important foundation to ensure that future medical professionals are not only aware of the ethical dimensions of AI in medicine but also equipped to make informed ethical decisions in their practice. Future research is required to develop problem-based and competency-oriented learning objectives and educational content for the proposed principle-based approach to teaching AI ethics in medical education.

## Introduction

### Background

Artificial intelligence (AI) and its applications have been of interest in both the scientific and societal domain for many years. AI has the potential to improve medical care through more accurate diagnosis and to reduce the burden on the health care system by reducing costs and workload [1,2]. Although AI in medicine has the potential to reduce the burden on medical staff, uncertainty about its capabilities raises concerns regarding job displacement [3]. The use of AI is expected to pose significant ethical challenges. AI algorithms are often trained on unrepresentative data, leading to potential discrimination and disadvantages for certain patient groups. Bias on the part of developers can also result in inequitable treatment [4]. The use of AI in medicine can also lead to erroneous diagnoses such as unnecessary treatment, which violates the basic principles of medical ethics [5].

Research recommends teaching AI ethics early in medical education to prepare for its potential impacts and challenges [6-8]. In addition to the technical and legal aspects of the use of AI in medicine, recent publications emphasize the importance of teaching AI ethics in medical education [9-11]. Recent studies have indicated that medical students anticipate significant ethical challenges from the use of AI in medicine [12,13]. Furthermore, research suggests limited knowledge and understanding of AI among medical students [14]. Despite the need for early teaching of AI ethics, there is a lack of guidance on specific content and methods for integrating AI ethics into medical curricula [10].

### Definitions of AI

Although the term *artificial intelligence* dates to the 1950s, there is inconsistency regarding its definition within the scientific community and the public [15]. On the basis of current scientific definitions, AI can be subdivided into “artificial general intelligence,” referred to as “strong AI” and “artificial narrow intelligence,” commonly referred to as “weak AI” [16]. Artificial general intelligence refers to the development of systems with “general intelligence,” capable of performing intellectual tasks comparable with humans. The term “artificial narrow intelligence” refers to an AI that has the capability to perform specific intellectual tasks comparable with humans without possessing general intelligence [17]. Artificial narrow intelligence can be subdivided into 2 main fields of current research: “symbolic AI” and “statistical AI.” On the basis of the idea of representing knowledge or certain intelligent behaviors using symbols and rules, “symbolic AI” commonly refers to rule-guided expert systems [16,18]. The term “statistical AI” refers to the development of systems that can find correlations and patterns within the analyzed data sets using statistical methods, without being explicitly programmed to do so or following predefined rules. Examples of “statistical AI” include “machine learning” (ML) with its subfield, “deep learning,” or “natural language processing” (NLP) [18]. While the ability to learn from data independently and increase their capabilities lies at the heart of ML, the subfield of deep learning focuses on the development of artificial neural networks that mimic the human central nervous system to process information. The subfield of NLP focuses on the analysis and processing of human language–based information by computer systems to enable improved human-computer interactions [16]. Advanced NLP techniques are, for example, used in large language models such as the AI-based chat applications available to the public, for example, ChatGPT (OpenAI, LLC) or Bard (Google LLC).

In medicine, AI and its respective subfields and specializations have attracted increased scientific interest in recent years [19]. For example, “symbolic AI” is used to develop rule-based expert systems such as “clinical decision support systems” (CDSSs) [20]. CDSSs aim to assist with diagnosis and selection of the best treatment for patients by providing information based on the current guidelines and information provided by experts. CDSSs follow rules and instructions predefined by experts and are therefore susceptible to ethical challenges such as the transfer of bias by experts or developers [21]. Because of their ability to analyze large amounts of data, systems based on ML are used to identify and process image-based data in medical specializations such as radiology or dermatology. An extensive study published in *Nature* in 2017 showed that systems based on ML are capable of detecting certain types of skin cancer (eg, malignant melanomas) with an accuracy comparable to that of dermatologists using image-based data [22].

As the data used to train ML-based systems and applications represent the basis of any subsequent analysis and therefore significantly influence accuracy, the data need to be representative of the target population [23]. This is especially important in the medical context, where demographic disparities in data can lead to systematic misdiagnoses or treatment recommendations that are less effective for underrepresented groups [24]. Unrepresentative data can potentially lead to bias and discrimination, with significant effects on patients [21,24]. To avoid any discrimination or negative effects for patients, the sources and composition of data sets used for AI development are of paramount importance. Ensuring the representation of the data is crucial, as the diversity and comprehensiveness of the data determine the system’s ability to generate reliable and valid outputs across different patient demographics [23]. Furthermore, acknowledging and addressing potential limitations and errors in AI products is essential for maintaining the validity of AI outputs, which directly affect the scope of their applicability in clinical settings [21]. AI systems trained on narrow or biased data sets may not only perform inadequately in diverse real-world scenarios but also misinform clinical decision-making, undermining the trust and credibility essential in medical practice [25]. The low accuracy and validity of AI, potentially leading to a lack of trust and credibility, could severely impact the utility of AI in the medical context. Utility refers to not only the performance of AI on a technological level but also how it translates into meaningful and practical advantages in health care settings. Therefore, the utility of AI in medicine is intrinsically linked to its ability to provide actionable, accurate insights that directly inform and enhance clinical decision-making [26]. It is therefore imperative to rigorously evaluate and validate AI systems against a variety of data sets that reflect the full spectrum of clinical cases and patient populations to ensure the utility, generalizability, and accuracy of AI tools in a broad range of health care contexts.

Although becoming broadly available rather recently, AI-based chat applications such as ChatGPT have rapidly emerged as significant tools with the potential to revolutionize various aspects of medicine, including the education and training of future physicians [27,28]. For example, these applications could be deployed for simulated patient-physician interactions, providing medical students with a low-risk environment to practice diagnostic skills and ethical decision-making [28]. The potential and broad availability of AI-based chat applications raise new ethical questions that necessitate comprehensive teaching in medical education.

### AI Ethics

The field of AI ethics was an area of interest for both scientific and governmental communities, even before the emergence of AI applications such as ChatGPT, which has gained widespread public attention [29,30]. However, there remains a lack of consensus on the definition of AI ethics, which can be attributed to several factors, including the novelty and interdisciplinary nature of the field as well as the absence of a widely accepted definition of AI [31].

Despite the current lack of consensus on the definition of AI ethics, some definitions are available. For example, AI ethics can be defined as “the emerging field of practical AI ethics, which focuses on developing frameworks and guidelines to ensure the ethical use of AI in society (analogous to the field of biomedical ethics, which provides practical frameworks for ethical practice in medicine)” [32]*.* This definition emphasizes the novelty of the field, further highlighting the importance of biomedical ethics.

The emphasis on biomedical principles is consistent with current scientific and governmental efforts aimed at developing AI ethics frameworks and guidelines to ensure the ethical development, deployment, and use of AI technologies [29,33]. The biomedical principles mentioned in the definition of AI ethics refer to the well-known and established principles of medical ethics initially proposed by Beauchamp and Childress [34]. The 4 principles of autonomy, beneficence, nonmaleficence, and justice are considered fundamental to medical ethics, while most guidelines and frameworks on AI ethics do not specifically focus on ethical considerations regarding the development, implementation, or use of AI in medicine; the emphasis on these principles further reinforces their importance [30,35].

Although existing guidelines and frameworks aim to address various ethical concerns related to AI, such as privacy, bias, accountability, and transparency, it should be noted that they fail to provide a clear definition of AI ethics [30]. Given the rapid pace of advancements in AI technology and its increasing impact on society, the need for clear and consistent definitions of AI and AI ethics is becoming increasingly urgent [30]. To specifically address ethical considerations related to AI in medicine and medical practice, a definition of “medical AI ethics” has been proposed, which “is an interdisciplinary subfield of AI ethics concerned with the application of ethical principles and standards to the research, development, implementation, and use of AI technologies within the practice of medicine” [10]. This definition emphasizes the importance of principles regarding the use of AI in medicine, which is fundamental to this study.

### AI Ethics in Medical Education

Although the need for teaching AI ethics in medical education is emphasized in scholarly literature, there is a lack of specification on relevant teaching content for AI ethics. In a recent scoping review, only a limited number of publications specifically focusing on the teaching of AI ethics as part of medical education were identified [10]. Although other publications acknowledge the importance of ethics in AI education, they do not provide specific content or guidance [36-39].

In one of the 2 identified publications specifically addressing AI ethics teaching content for medical education, 6 potential topics were defined: informed consent, bias, safety, transparency, patient privacy, and allocation [9]. The 6 teaching subjects were proposed to address the potential challenges related to the application of AI in medicine. For example, the anticipated challenge of *informed consent* highlights the importance of patient autonomy, potentially impeded by the lack of transparency or explainability in the decision-making of AI-based applications. Besides these 6 potential teaching subjects, the importance of teaching fairness and responsibility is emphasized by another publication that focuses on AI ethics education [11]. Furthermore, the importance of empathy has been emphasized in relation to the use of AI in medicine and the associated need to teach AI ethics [6].

A recurrent theme related to the teaching of AI ethics as part of medical education focuses on the principles of medical ethics according to Beauchamp and Childress (autonomy, beneficence, nonmaleficence, and justice) [10]. This emphasis is also echoed by existing guidelines and frameworks regarding AI ethics [30]. Additional recommendations on AI ethics teaching content include “explainability,” “liability,” and “accountability,” which are also considered important by available guidelines [30,40]. On the basis of the analysis of existing publications on teaching AI ethics in medical education, 12 potential subjects were considered for teaching AI ethics. The 12 identified potential teaching subjects for AI ethics in medical education are listed in Textbox 1.

**Recommended artificial intelligence (AI) ethics teaching content with specific descriptions.**

**Informed consent**

Informed consent in the context of AI in medicine requires that patients be fully informed about treatment options and risks, necessitating a comprehensive understanding and explanation of AI technologies by physicians.

**Bias**

The use of AI in medicine may exhibit biases stemming from nonrepresentative data or structural conditions, leading to potential discrimination based on sex, age, or socioeconomic status.

**Safety**

The use of AI in medicine can have potentially harmful consequences for patients, necessitating a critical examination of the accuracy of AI-based applications and clear communication of their limitations.

**Transparency**

Transparency in AI-based medical applications is essential for understanding decision-making processes, influencing the quality and ethics of patient care, and maintaining trust, particularly in critical scenarios.

**Privacy**

Privacy not only refers to implementing technical data protection measures but also comprehensively understanding the ethical implications of handling sensitive patient data.

**Allocation**

In the context of AI in medicine, allocation refers to equitable access to technology and the impact of AI on equitable access to care.

**Fairness**

Fairness in AI ethics within medicine refers to ensuring equitable treatment for all patients regardless of their background. This encompasses the need for AI systems to be free from biases that may affect diagnosis, treatment recommendations, or patient outcomes.

**Responsibility**

Responsibility in the context of AI ethics in medicine emphasizes the importance of health care professionals and AI developers to using AI tools responsibly. This includes ensuring that these tools are safe, reliable, and used in a manner that benefits the patients.

**Empathy**

Empathy in the context of AI underscores the importance of maintaining the human aspect of health care, especially as AI technologies become more prevalent.

**Explainability**

Explainability in AI in medicine is closely linked to transparency and is important for understanding the AI-based decision-making process, affecting physician-patient relationships, and shared decision-making.

**Liability**

Liability in medical AI ethics concerns the potential for treatment errors related to the use of AI in the medical context. Questions on liability extend from potential users to health care institutions and AI developers.

**Accountability**

Accountability in medical AI involves understanding the associated limitations and competent oversight by medical professionals. This includes critically assessing AI errors and biases and ensuring accurate, informed, and ethical applications within medical decision-making. In addition, this accountability extends to continuously monitoring AI performance and adapting to evolving ethical and clinical standards in medical practice.

### Objective

On the basis of a discussion and reflection theoretical analysis of the recommended teaching subjects on AI ethics informed by existing literature (as specified in the AI Ethics in Medical Education section), this study aims to introduce a set of ethical principles for “medical AI ethics.” As the proposed AI ethics teaching subjects for medical education in the existing scientific literature primarily focus on the challenges associated with the use of AI in medicine, they fail to acknowledge the broader implications of foundational ethical principles. By concentrating on a principle-based approach to AI ethics, this paper aims to address the gap in the existing scientific literature, serving as a foundational framework for AI ethics teaching content in medical education.

### Theoretical Analysis of Recommended AI Ethics Teaching Subjects in Medical Education

#### Overview

Ethics commonly relies on principles as foundational guidelines for decision-making and behavior. The 4 foundational principles of medical ethics—autonomy, beneficence, nonmaleficence, and justice—are highly relevant in the context of teaching ethics in medical education [41].

While these 4 principles have been an integral part of current scientific publications on AI ethics in medical education, the recommended teaching subjects are mainly derived from the anticipated challenges associated with the use of AI in medicine [10]. Addressing these challenges is important for fostering a comprehensive understanding regarding the use of AI in medicine. However, this approach does not fully capture the multidisciplinary and interdisciplinary nature of this field. The complexity of AI ethics in medicine extends beyond these anticipated challenges, encompassing a wide range of disciplines such as law, medicine, ethics, and computer science. For example, the proposed teaching subject of “informed consent” warrants a detailed analysis to exemplify the high level of interdisciplinarity present in AI ethics, intersecting with each of the other proposed teaching subjects. This interconnection results in a substantial overlap, which can challenge the establishment of clear distinctions between the different areas of AI ethics.

The methodology of this study is anchored in a theoretical approach, building upon a previous comprehensive scoping review of the existing literature on teaching AI ethics in medical education [10]. This also includes the consideration of relevant guidelines and frameworks regarding the ethics of AI, resulting in the identification of 12 potential teaching subjects for AI ethics as detailed in Textbox 1. To exemplify the high level of interdisciplinarity present in AI ethics by focusing on the subject of “informed consent,” the publications included in the scoping review, including the proposed challenges associated with the use of AI in medicine, were re-evaluated. This theoretical analysis provides the foundation for the development of the principles of medical AI ethics presented in the Medical AI Ethics section. The theoretical basis of the proposed principle-based approach to AI ethics is further strengthened by our expertise as we specialize in the ethical use of AI in medical and public health contexts. This background informs the depth and rigor of the analysis, ensuring that the developed framework is both relevant and grounded in practical ethical considerations in these fields. The theoretical methodology we used is characterized by a focus on conceptual development and theoretical insights rather than empirical testing or data collection.

#### Informed Consent

##### Overview

Informed consent represents an important development in medical ethics and patient rights, representing a departure from the historically paternalistic nature of medical practice [42]. In earlier medical paradigms, decision-making was predominantly physician driven, with minimal patient involvement. This approach, often paternalistic, assumes the primacy of the physician’s judgment, potentially leading to interventions conducted without comprehensive patient understanding or consent [42].

The development and integration of informed consent into medical practice represents a substantial cultural and ethical transition toward acknowledging and upholding patient autonomy. Central to this evolution is the concept of shared decision-making (SDM), a collaborative process that involves physicians and patients jointly making treatment decisions. SDM encompasses a thorough discussion of available treatment options, including their benefits and risks, and considers patient values, preferences, and circumstances [42,43]. This method positions patients as active participants in their health care journey rather than as passive recipients of medical decisions.

In this context, informed consent is pivotal in facilitating SDM, as it ensures that patients are not only informed of their medical choices but also engaged in selecting options that resonate with their personal health goals and values. This approach transforms the traditional physician-patient relationship into a partnership, where decisions are mutually agreed upon, thereby honoring the patient’s right to self-determination. It also fosters a deeper level of trust and respect within the physician-patient relationship.

As a result, informed consent serves more than just a legal requirement to minimize liabilities; it is a crucial aspect of patient-centered care and a fundamental element of ethical medical practice. This signifies the transition from a paternalistic approach to one that emphasizes patient autonomy and upholds the principles of SDM.

##### Informed Consent in the Context of AI in Medicine

Regarding the development, implementation, and use of AI in medicine, the concept of informed consent warrants a comprehensive introduction owing to the technical complexities inherent to AI. AI systems, particularly those used in diagnostics and treatment recommendations such as ML, often involve algorithms that might be nontransparent to both patients and health care professionals. This lack of transparency presents a substantial challenge to the conventional process of informed consent, complicating the task of understanding and communicating how an AI-based application formulates recommendations [44].

Moreover, the development of AI-based applications involves extensive data sets, raising concerns regarding data privacy and the potential for expropriation of personal health data [9]. These issues necessitate clear communication with patients throughout the physician-patient relationship and during the process of ensuring informed consent. It is imperative that patients are adequately informed about not only the advantages and risks associated with AI-assisted treatments but also the manner in which their data are used, protected, and stored [45]. With the increasing integration of AI in medicine and health care, the process of obtaining informed consent must be adapted to meet these challenges, thereby ensuring that patients retain control over their health care decisions in an environment increasingly influenced by AI.

#### Intersections of Informed Consent With Key AI Ethics Teaching Subjects

##### Overview

This section aims to underscore interdisciplinarity and intersectionality among the recommended teaching subjects in AI ethics, as outlined in the Informed Consent section, with informed consent serving as a representative example. Focusing on these intersections, this section highlights the importance of an integrated educational approach in the context of medical AI ethics. Such an approach acknowledges that topics such as bias, privacy, and transparency, among others, are not merely isolated subjects but instead require a comprehensive, holistic evaluation. Embracing this integrated perspective is important for a comprehensive understanding of AI ethics in medical practice and education, underscoring the need to re-evaluate and potentially refine current teaching recommendations. To effectively illustrate the interdisciplinarity and interconnectedness of frequently recommended teaching subjects for AI ethics in medical education, “informed consent” should be discussed in the context of 5 frequently proposed teaching subjects: bias, safety, transparency, privacy, and liability.

##### Bias

To enable patients to make informed decisions when AI-based applications are used in their treatment, it is important to address the possibility of bias inherent in these technologies. Informed consent in this context requires the awareness and understanding of potential biases in AI decision-making processes [46]. For instance, a diagnostic AI-based application might exhibit varying levels of accuracy across different demographic groups, potentially owing to data representation issues [21]. Patients must be informed of such disparities in accuracy as this information is vital for them to consent to the use of AI in their treatment.

##### Safety

The safety of AI-based applications in medicine is a critical component of informed consent for medical treatment recommendations involving AI. Patients must be clearly informed about the potential risks associated with AI-driven medical decisions, including the possibility of erroneous outcomes such as false positives or negatives [47]. This comprehensive understanding of the safety profile of AI-assisted treatments is essential for patients to make informed decisions about their care. Being informed and knowledgeable about the limitations and risks of AI technologies ensures that patients can weigh these factors against potential benefits when consenting to AI use in their treatment.

##### Transparency

Transparency in AI systems is important not only for patients but also for physicians, who serve as the primary receivers and communicators of AI-driven medical information. A clear understanding of how AI-based applications work, particularly how decision-making processes are performed, is required for physicians to effectively communicate with their patients [48]. Such informed communication is a fundamental aspect of the informed consent process, fostering a deeper understanding and trust within the physician-patient relationship [49]. When patients receive comprehensive and transparent information from their trusted health care providers, they enhance their engagement and participation in decision-making. Therefore, transparency in AI goes beyond technical clarity and is crucial for fostering a strong physician-patient relationship, ensuring that informed consent is based on a shared understanding of the potential risks and benefits associated with AI-assisted treatments [50].

##### Privacy

The process of obtaining informed consent for AI-based medical treatment recommendations should include data privacy. It is important for patients to be informed about the use, access, and protection of their data. Ensuring that patients understand how their personal health data are used, who has access to it, and the measures in place to protect it is a key component of the informed consent process [51]. This comprehensive disclosure and transparency regarding data handling are vital for maintaining the integrity of the physician-patient relationship and for upholding the ethical standards of medical practice in the era of AI.

##### Liability

Regarding the use of AI in medicine, it is imperative to address the concept of liability in the informed consent process. Patients should be clearly informed of the potential for errors and liability issues associated with AI-driven medical decisions [52]. This conversation should entail a discussion on who bears responsibility, including the liability of physicians, if an AI system malfunctions or leads to incorrect medical outcomes such as misdiagnoses or inappropriate treatment plans. The explicit clarification of liability, particularly the role and responsibility of health care providers in conjunction with AI, is important for helping patients understand the potential risks involved [53]. This understanding is a key component of a comprehensive informed consent process that directly affects the patients’ trust in AI and their treating physicians. By transparently addressing these liability concerns, including the physicians’ responsibilities, health care providers can reinforce the integrity of the physician-patient relationship and uphold the ethical standards of medical practice in an AI-integrated health care environment [53].

### Medical AI Ethics

#### Overview

The high degree of interdisciplinarity and intersectionality in AI ethics, as detailed in the previous section, highlights potential conflicts in teaching AI ethics based solely on the anticipated challenges associated with the implementation and use of AI in medicine. This complexity underscores the necessity of adopting a principle-based approach to AI ethics education, mirroring established pedagogical frameworks in medical ethics education [41].

In the context of traditional medical ethics education, the emphasis on foundational principles provides a broad and adaptable framework that is essential for understanding and addressing complex ethical dilemmas. This approach facilitates the holistic comprehension of ethical issues, offering the flexibility to accommodate the diverse and evolving nature of medical scenarios. Similarly, when considering AI in medicine, a focus on core ethical principles rather than solely on specific challenges lays the groundwork for a robust and comprehensive educational strategy. Future medical professionals should be equipped with a deeper and more nuanced understanding of ethical decision-making by emphasizing ethical principles in the context of implementing and using AI in medicine. This principle-based approach ensures that medical ethics education remains relevant and responsive to the dynamic landscape of AI integration in medicine. The goal is for medical students to be able to effectively navigate the ethical complexities associated with AI technologies in medicine, not just focusing on potential challenges but also emphasizing the ethical values that are essential to medical practice.

Owing to the paramount importance and relevance of the 4 principles of medical ethics formulated by Beauchamp and Childress [34], the principles of autonomy, beneficence, nonmaleficence, and justice should provide the essential foundation for medical AI ethics. These 4 principles are subsequently introduced based on existing scholarly discourse, focusing on the use of AI in medicine, with an emphasis on medical education.

Traditional medical practices have predominantly focused on individual relationships between physicians and patients. However, modern health care increasingly necessitates considering broader aspects such as cost-effectiveness, resource allocation, and proportionality, especially in light of financial constraints. A prominent illustration of these evolving dynamics in medical practice is the COVID-19 pandemic. This global health crisis underscored the critical importance of public health considerations and highlighted extensive interdisciplinarity and interconnectivity within the field of medicine. The COVID-19 pandemic has highlighted the importance of balancing individual patient care with broader public health measures [54]. It demonstrated how medical decisions are not made in isolation but are profoundly influenced by factors such as resource availability, health care infrastructure, and broader societal implications. This scenario emphasizes the crucial role of public health principles in informing medical practices, particularly in crises. The pandemic also illustrates the necessity of integrating insights from various disciplines, including epidemiology, health economics, and ethics into medical decision-making.

Given the anticipated impact of AI on the field of medicine, which extends beyond the traditional concept of medical practice owing to its inherent interdisciplinarity and complexity, ethical considerations must be adapted accordingly. The scope of AI in medicine introduces novel ethical dimensions that require a broader framework for ethical analysis. Therefore, the integration of 3 principles of public health ethics—efficiency, common good orientation, and proportionality—is proposed along with the established principles of medical ethics to form a comprehensive foundation for medical AI ethics [55-58].

**Figure 1:**
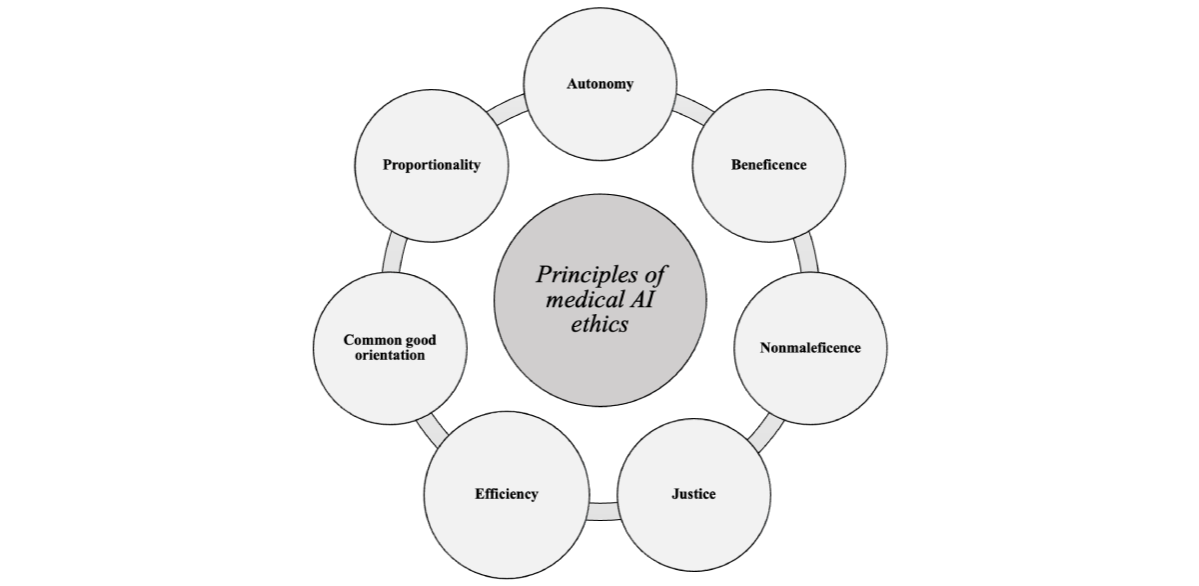
The principles of medical artificial intelligence (AI) ethics for medical education.

Similar to the principles of medical ethics outlined by Beauchamp and Childress [34], each principle of public health ethics is examined in subsequent sections with a specific focus on its relevance to AI in medical practice and education. While the principles of public health ethics may not be as established or universally agreed upon as those of medical ethics, their inclusion provides a suitable framework to address the unique challenges posed by AI in medicine and health care. This extended ethical framework aims to provide a more comprehensive understanding of the role and implications of AI in medicine, ensuring that future medical professionals are equipped to make ethically sound decisions in increasingly AI-integrated medical practice. The proposed principles of AI ethics for medical education are presented in Figure 1.

#### Autonomy

The principle of autonomy in medical ethics emphasizes the right to make independent decisions regarding health care [34]. This principle recognizes an individual’s capacity for self-determination and personal choice, affirming that patients have the authority to provide or withhold consent for medical treatment. Respecting autonomy in medical practice involves providing patients with sufficient information, ensuring comprehension, and facilitating independent decision-making [59]. This respect for autonomy is closely tied to the principle of informed consent, which ensures that patients actively participate in decisions regarding their care and treatment.

In the context of using AI in medicine, particularly in diagnostics and treatment recommendations, technology introduces new challenges and opportunities to maintain patient autonomy [60]. For example, when using AI-based diagnostic applications, it is crucial to inform patients about how these tools impact their health care decisions, ensuring that informed consent is comprehensive. Equally important is equipping physicians with the knowledge to balance AI-generated insights with their clinical expertise, thus upholding both patient and physician autonomy in decision-making processes. The incorporation of AI into health care decision-making can affect the presentation and comprehension of options by patients. Ensuring that patients retain their autonomous decision-making power in an AI-driven environment requires the careful consideration of how the information is communicated and understood [60]. Autonomy in this context extends to ensuring that patients have a clear understanding of AI interventions and their capabilities, limitations, and impact on personal health decisions. Moreover, the principle of autonomy extends to physicians. If AI increasingly assists in medical decision-making, it is imperative that physicians remain empowered to make independent professional judgments, balancing AI insights with their clinical expertise and ethical considerations.

The principle of autonomy addresses several anticipated challenges and recommends teaching subjects on AI ethics in medical education. For example, in the context of informed consent, autonomy ensures that patients are fully aware of the role and limitations of AI in their treatment, including potential bias and safety concerns. Autonomy also involves clear communication regarding data privacy, ensuring that patients understand how their data are used in AI systems. In the context of using AI in medicine, autonomy is not limited to the patient’s understanding and decision-making; it also encompasses the physician’s ability to make independent judgments informed by, but not solely reliant on, AI-driven data. This dual focus preserves the integrity of clinical decision-making and respects both the patient’s and the physician’s autonomous roles. Furthermore, transparency and explainability in AI systems are fundamental to ensure that patients autonomously understand and evaluate AI-driven health care choices. Autonomy acts as a guiding principle that addresses these challenges, ensuring that patient rights and self-governance remain central to the increasingly AI-integrated landscape of medical practice. This principle also extends to the equitable allocation of medical resources and fairness in treatment decisions, where an autonomous choice must be informed by unbiased AI recommendations. This comprehensive approach to autonomy in AI ethics education underscores the need for a balanced consideration of both patient and physician perspectives to ensure ethical integrity in the application of AI in medicine.

#### Beneficence

The principle of beneficence, a fundamental aspect of medical ethics, underscores the responsibility of health care providers, including physicians, to act in the best interests of patients [61]. This principle is the basis of the ethical framework guiding health care delivery and promoting actions that enhance patient well-being and welfare [34]. In medical practice, beneficence guides physicians to consider the actual benefits of medical interventions, extending from the sole minimization of potential harm. Therefore, this principle encompasses a broader responsibility toward enhancing the overall quality of life of the patient, affirming that every medical decision should contribute positively to the holistic well-being of the patient [50].

The principle of beneficence is paramount in the application of AI in medicine, such as through predictive analytics and personalized medicine. Although promising, AI-based applications must be critically evaluated for their efficacy and safety to ensure alignment with the overarching goal of promoting patient well-being, which reflects the true essence of beneficence in medical practice [62]. In addition, it is crucial to ensure that AI-based applications align with the broader goals of patient care, emphasizing not only clinical outcomes but also patient quality of life and overall well-being. Such an approach should consider individual social backgrounds and personal circumstances, ensuring that AI-driven health care focuses on the diverse needs of each patient [50].

In the context of AI ethics and medical education, beneficence emphasizes the importance of developing, implementing, and using AI applications designed with the primary aim of improving patient outcomes. This includes addressing potential biases in AI algorithms that could negatively impact patient care, ensure patient safety, and maintain transparency in the AI decision-making processes. Therefore, the principle of beneficence guides the ethical application of AI in medicine, ensuring that these advancements aim to maximize patient benefits and well-being, consistent with the overarching goals of medical practice.

#### Nonmaleficence

Although the principle of nonmaleficence also focuses on ensuring the best possible treatment for patients and aligning all actions accordingly, it emphasizes that health care professionals should do no harm [34]. This principle is complementary to the principle of beneficence, and it aims not only to prevent harm but also to proactively avoid and reduce risks associated with medical care. Nonmaleficence requires that the risks of any medical intervention are carefully weighed against their potential benefits and actions that could cause harm are avoided. This principle underlines the responsibility of health care providers to ensure that any treatment or medical advice does not adversely affect a patient’s health.

The potential risks of using AI in medicine, such as misdiagnosis, algorithmic biases, and data security breaches, reinforce the relevance of the principle of nonmaleficence. To ensure nonmaleficence, the rigorous testing and validation of AI systems, ongoing monitoring for adverse outcomes, and commitment to addressing any safety concerns are crucial [62]. Moreover, this commitment extends to the ethical development and deployment of AI technology. It involves actively working to mitigate risks, such as biases in training data, that could lead to unequal or unfair treatment outcomes [50].

To raise the awareness of potential conflicts with the principle of nonmaleficence regarding the use of AI in medicine, medical education should focus on the ethical design, development, and deployment of AI applications in medicine. Therefore, nonmaleficence is an important part of medical AI ethics, emphasizing the need to ensure the accuracy and reliability of treatment recommendations originating from the use of AI-based applications in medicine. Teaching content on nonmaleficence addresses various anticipated challenges regarding the use of AI in medicine, such as safety, privacy, bias, and transparency. By adhering to the overarching principle of nonmaleficence, physicians can navigate the ethical challenges posed by AI in medicine, ensuring that the technology is used in ways that prioritize patient safety and harm reduction.

#### Justice

The principle of justice in medical ethics, as outlined by Beauchamp and Childress [34], is concerned with ensuring fair and equal treatment for all patients regardless of their socioeconomic status, background, or circumstances. This principle emphasizes the importance of fairness in the distribution of resources and access to health care services. In practical medical settings, justice can be translated into unbiased decision-making, equal opportunity for treatment, and eradication of any form of discrimination.

Justice is an important aspect regarding the use of AI in medicine. Owing to the risk of bias due to unrepresentative training data, for example, treatment recommendations from the use of AI in medicine could lead to disadvantages for different groups or individuals, directly conflicting with the principle of justice [50]. Furthermore, access to the technology itself could be limited, for example, by economic means, thereby potentially perpetuating existing inequalities in access to advanced medical technologies [35]. This potential for injustice can be further exacerbated if an increasing prevalence of AI in medical practice is anticipated.

Owing to the substantial risk of injustice with the use of AI in medicine, medical education should include teaching the principle of justice in the context of AI. Focusing on the equitable availability and use of AI technologies, future physicians should be trained to recognize and address the potential inequities that AI might introduce or perpetuate. Therefore, teaching the principle of justice, extending from traditional medical ethics education, can serve as a foundation to address anticipated challenges such as allocation, bias, fairness, liability, and accountability. For instance, when considering liability and accountability, justice refers to ensuring that patients are not disproportionately affected by errors or failures in AI systems. It involves advocating for systems that hold developers and health care providers responsible for potential technological malfunctions, ensuring that accountability measures are in place to protect all patients from potential harm or injustice, especially those in vulnerable or marginalized groups [53].

#### Efficiency

Efficiency within public health ethics underscores the strategic use of resources to maximize health benefits for the population [57]. This principle is not only solely an economic concern but also a moral imperative to ensure the equitable and judicious use of medical technologies and services. Ethical considerations regarding the principle of efficiency are especially relevant in health care settings where resources are limited and demand is high, as exemplified in the context of the COVID-19 pandemic.

Owing to the capabilities of AI in medicine with the potential to enhance the efficiency of medical services through faster and more accurate diagnostics, it is crucial to consider the ethical implications of these developments [19]. The ability of AI to rapidly analyze large data sets can greatly enhance the speed of diagnostic procedures, which could result in more timely patient care and improved treatment choices that are more precise. However, this benefit is contingent on the data quality. Poor-quality data can result in AI models that incorrectly predict outcomes based on artifacts in the data rather than actual clinical results [21]. Therefore, the ethical use of AI in health care must include rigorous validation of the data quality to ensure accurate and reliable outcomes. For example, physicians must balance the efficiency gains offered by AI with the need for clinical judgment and personalized patient care and upholding and maintaining the quality of physician-patient relationships [63].

Teaching the principle of efficiency in the context of AI ethics education should focus on the balance between technology-driven efficiency and patient-centered care. Future physicians need to understand how to leverage AI to optimize health care delivery without compromising quality of care. Therefore, teaching the principle of efficiency highlights the anticipated challenges related to a lack of empathy. It is imperative to ensure that the pursuit of efficiency through AI does not lead to the depersonalization of patient care. Empathy remains a crucial aspect of health care, and AI systems should be used to enhance, rather than replace, the human elements of patient interaction and care.

#### Common Good Orientation

Common good orientation is a guiding principle of public health ethics, aiming to improve the collective well-being and health of the community or population as a whole [58]. This principle extends the focus of individual patients, emphasizing the interconnectedness between individual and public health. This involves considering the wider impacts of health care interventions and prioritizing actions that promote the health and welfare of the public.

The principle of common good orientation in the context of AI, crucial in guiding the integration of technology into medical practice, calls for a delicate balance between individual patient benefits and the collective well-being of the community. It is essential to recognize how AI in medicine can address or potentially exacerbate health disparities [64]. The ability of AI to process and analyze data can be harnessed to identify and address gaps in health care delivery, offering insights into underserved populations and tailoring interventions to meet their specific needs. Conversely, if not carefully managed, AI could unintentionally increase these disparities by favoring populations with better access to the technology. This duality underscores the need for AI advancements in health care to contribute positively and equitably to public health, promoting fairness in health care access and outcomes. It is important to note that the selective application of AI not only undermines the principle of common good orientation but also risks creating a perception of elitism in the medical profession. Such a scenario could harm the reputation of the medical field, rendering it as unevenly benefiting certain populations. Furthermore, using AI in medical practice could potentially lead to events where patients are harmed, for example, through biased decision-making or errors made by users. This could potentially lead to a negative perception of AI within the broader population, which in turn may result in a general unwillingness or resistance to adopting AI technologies. This hesitance could directly conflict with the principle of common good orientation, as it hinders the widespread and equitable implementation of AI that could benefit the entire community [25].

Teaching the principle of common good orientation in the context of AI ethics in medical education underscores the importance of developing, implementing, and using AI technologies in ways that serve a wider community not just the individual patient. This includes understanding the potential of AI in managing public health crises such as pandemics. Medical education based on the principle of common good orientation emphasizes aspects of safety, transparency, allocation, and responsibility, which are important to best prepare for potential challenges through AI in medicine and associated ethical considerations.

#### Proportionality

The principle of proportionality in public health ethics necessitates a balanced approach to medical interventions that weighs benefits against risks [57]. Therefore, this principle can be applied to ensure that the measures taken, such as medical interventions, are proportional to the health risks that they aim to mitigate. In medicine, proportionality is important in decision-making, ensuring that the intervention aligns with the expected health outcomes.

In medical practice, the principle of proportionality is important when considering the integration of AI technology to balance benefits against potential risks for individual patients and the broader population. This principle necessitates a careful assessment of the role of AI, particularly in ensuring equitable resource distribution and maintaining public trust [25]. For instance, when using AI for diagnostics, it is necessary to evaluate the accuracy and effectiveness of the technology against risks, such as misdiagnosis or overreliance on AI. This evaluation should consider not only the immediate impact on individual patients but also the broader implications for health care resources and community trust. In the critical area of resource allocation within health care, the use of AI holds substantial promise in enhancing the efficiency and effectiveness of distributing limited medical resources [63]. However, it is essential to guard against the risk of AI systems inadvertently perpetuating existing biases or failing to address the diverse needs of different patient groups. This calls for a transparent, community-engaged approach to the development and deployment of AI in health care, ensuring that AI recommendations do not unfairly disadvantage any patient group [24]. By adhering to the principle of proportionality, health care providers can better navigate the ethical complexities of using AI, ensuring that its application is not only technologically sound but also ethically responsible, both at the individual patient level and in the wider context of public health.

The principle of proportionality can be helpful for future physicians to comprehend the anticipated challenges of AI in medicine, particularly regarding the aspects of allocation. This principle also addresses other anticipated challenges such as transparency and explainability to understand how decisions are made and whether the overall population is considered, ensuring that recommendations are reasonable.

## Discussion

### Overview

The integration of AI in medicine necessitates a nuanced approach to ethics education that addresses the unique challenges and opportunities introduced by this technology. By exploring public health and medical ethics principles, medical AI ethics offers a comprehensive framework for guiding future physicians in this complex landscape. The proposed teaching of medical AI ethics in medical education emphasizes the importance of ethical principles rather than focusing solely on anticipated challenges, aiming to foster a deeper understanding of potential ethical considerations and enable adaptation in the light of rapid technological advancements.

Given the dynamic nature of AI and the associated rapid technological advancements, for example, as demonstrated by AI-based chat applications such as ChatGPT, ethical considerations need to be continually adapted [65]. The need for timely adaptation challenges traditional ethics education in medicine, which may not account for the current use of AI in medicine. Traditional ethics education primarily focuses on the 4 principles of medical ethics as formulated by Beauchamp and Childress [41]. While these principles can provide valuable guidance in the age of AI in medicine and are therefore foundational to the proposed medical AI ethics education, adaptation is needed to reflect the complexities and challenges introduced by the implementation and use of AI in medicine and medical practice.

The high level of intersectionality and interdisciplinarity inherited by the implementation and use of AI in medicine highlights the importance of a principle-based approach rather than solely focusing on anticipated challenges. While the proposed ethical principles also show a high level of interconnectivity, the chosen educational approach aims to encourage a more nuanced understanding, not limited to specific anticipated challenges but rather to enable future physicians to adapt to the changing landscape associated with the use of AI in medicine, facilitating the consideration of multiple ethical dimensions simultaneously. In addition to the proposed principles, medical education should incorporate practical case studies and simulations to reflect real-world scenarios. For example, applying AI to patient triage during health emergencies such as the COVID-19 pandemic can offer practical contexts for students. This approach would not only enhance their understanding of ethical principles but also prepare them for decision-making in complex, real-life medical situations influenced by AI. It is important for future physicians to understand the balance between the potential benefits of AI and the ethical implications of its use, particularly in scenarios in which biased algorithms could lead to unequal treatment of diverse patient groups. Therefore, a comprehensive curriculum that includes both theoretical knowledge and practical applications is essential to cultivate ethically informed medical professionals.

An in-depth and interdisciplinary understanding of ethics is important in the dynamic field of medical AI. This importance is underscored by the fact that the integration of AI into medical education may not always keep pace with rapid advancements in medical practice. A focus on ethical principles rather than solely on specific challenges of AI use in medicine aims to prepare medical students for various scenarios in the medical context. This approach maintains relevance even if the AI applications used in education are not representative of the latest state-of-the-art developments in medical AI. The principle-based approach to AI ethics offers broader applicability and reduces dependence on the most recent AI technologies, potentially benefiting medical schools with limited financial resources. In addition, AI products for teaching, often sourced from third parties and guided by cost considerations, may pose unique challenges such as the risk of bias or rapid obsolescence [66,67]. This necessitates awareness, among medical students, of the potential ethical issues associated with these tools. By emphasizing a principle-based approach to AI ethics, educators can equip students with the necessary understanding to navigate the evolving landscape of AI in medicine, fostering adaptability and ethical sensitivity in future medical professionals. This adaptability is crucial to ensure that future physicians are prepared for the ethical dilemmas they may encounter in a rapidly evolving AI landscape.

In the applicability of the principle-based approach to AI ethics, the paramount importance of AI-based chat applications such as ChatGPT must be assumed [68]. As ChatGPT demonstrated extensive medical knowledge, as exemplified by its ability to pass the written part of the United States Medical Licensing Exam, AI-based chat applications offer new opportunities for medical education and medical students, such as in simulated patient interactions and case study analysis [69,70]. However, as ChatGPT was not explicitly developed for use in the medical context and, for example, does not adhere to stringent medical device regulations, it raises new ethical challenges. This becomes particularly evident, as AI-based chat applications can hallucinate and might not provide correct medical information due to improper “prompting” [70]. The limitations of ChatGPT, such as inaccurate or misleading medical information, necessitate an awareness of not only the technical limitations but also the associated ethical considerations. This reinforces the importance of a principle-based approach to AI ethics in medical education, emphasizing the importance of critically reflecting on and evaluating any use of AI in medicine. Awareness of potential ethical considerations regarding AI-based chat applications also extends from the provision of medical knowledge to a broader medical context, such as scientific research [71]. For example, if AI-based chat applications such as ChatGPT are used for medical research, medical education should facilitate an understanding of how this could impact research integrity or potentially interfere with the existing ethical standards [71]. Medical education should prepare students to navigate through these complexities, ensuring the ethical integration of AI in practice and research.

Although the integration of public health ethics principles as part of medical AI ethics offers a comprehensive approach for teaching AI ethics in the medical setting, it is important to recognize that the field of public health ethics is still evolving [72]. Unlike the well-established principles of medical ethics proposed by Beauchamp and Childress [34], public health ethics principles such as efficiency, common good orientation, and proportionality are not universally agreed upon or applied consistently across different contexts. This lack of standardization presents a challenge for formulating a universally applicable ethical framework for AI in medicine. Furthermore, the interdisciplinary nature of public health ethics, encompassing the aspects of sociology, economics, and political science, adds to the complexity of integrating these principles into medical AI ethics education. This complexity requires careful consideration during curriculum development to ensure that these principles are taught in a manner that is both relevant and applicable to medical students. Moreover, the rapidly changing landscape of AI technology necessitates a dynamic approach to ethics education in which principles and guidelines are continuously revisited and updated. This need for adaptability may challenge the traditional formats of medical education, calling for innovative pedagogical approaches to ensure that future physicians are adequately prepared for the ethical complexities of AI-integrated medical practice.

### Limitations

This study and the proposed theoretical foundation to medical AI ethics is subject to several limitations that need to be considered. Continuous evolution in the field of AI presents substantial challenges for the development of static ethical guidelines and frameworks for medical education. The dynamic nature of AI technology underscores the need for an adaptable and responsive ethical framework in medical education, particularly in the context of public health ethics, where principles are still developing and gaining consensus. Given that new advancements, for example, as exemplified by AI-based chat applications such as ChatGPT, cannot be foreseen and that the capabilities of AI and AI-based applications in medicine are anticipated to expand, continuous updates of existing educational frameworks and content are required.

Furthermore, the applicability and relevance of ethical principles as a part of medical AI ethics education may vary across cultural and health care settings. Different regions may have varying access to AI technologies, and cultural values may influence the perceptions of integrating and using AI in the medical setting. This variability could impact the universality of the proposed ethical framework and limit the applicability of teaching medical AI ethics as a part of medical education.

Moreover, integrating new teaching content into medical curricula is challenging due to the need for time-intensive accreditation processes and extensive teaching content. The integration of new teaching content such as medical AI ethics education requires careful planning to ensure that future physicians are adequately prepared and not overwhelmed by information. In addition, limited access to instructors knowledgeable in ethics, medicine, and AI may pose a challenge to implementing the proposed teaching of medical AI ethics, as these experts may not be available in most institutions.

### Conclusions

This study highlights the imperative need for medical AI ethics education and the integration of a comprehensive set of ethical principles into medical education to prepare physicians for the ethical challenges posed by AI in medicine. As the advancement of AI technologies in medicine is expected to increase, it is essential for medical ethics education to adapt and evolve accordingly to keep pace with these developments. Educational institutions should take proactive steps to update their curricula, ensuring that future medical professionals are not only aware of the ethical dimensions of AI in medicine but also equipped to make informed ethical decisions in their practice. The principles discussed, drawn from both traditional medical and public health ethics, provide a multidimensional framework for understanding and navigating the ethical landscape associated with the use of AI in medicine.

Given the rapid advancements in the field of AI, it is essential that these ethical guidelines be regularly revisited and updated to remain relevant in the context of medical education. The proposed dynamic approach, with an emphasis on ethical principles, aims to ensure that medical professionals not only are equipped to use AI in ways that enhance patient care but also uphold the highest ethical standards. Future research is needed to develop problem-based and competency-oriented learning objectives and educational content for medical AI ethics and implementation and validation.

## Abbreviations

AI: artificial intelligence
CDSS: clinical decision support system
ML: machine learning
NLP: natural language processing
SDM: shared decision-making
